# New Instrumented Trolleys and A Procedure for Automatic 3D Optical Inspection of Railways

**DOI:** 10.3390/s20102927

**Published:** 2020-05-21

**Authors:** Maria Cristina Valigi, Silvia Logozzo, Enrico Meli, Andrea Rindi

**Affiliations:** 1 Department of Engineering, University of Perugia, 06125 Perugia, Italy; sililog@hotmail.com; 2 Department of Industrial Engineering, University of Florence, 50139 Florence, Italy; enrico.meli@unifi.it (E.M.); andrea.rindi@unifi.it (A.R.)

**Keywords:** automated inspection systems, rail inspection, 3D wear maps, 3D optical scanners

## Abstract

This paper focuses on new instrumented trolleys, allowing automated 3D inspection of railway infrastructures, using optical scanning principles and devices for defects and damage evaluation. Inspection of rolling components is crucial for wear evaluation and to schedule maintenance interventions to assure safety. Currently, inspection trolleys are mainly instrumented with 2D contact or optical sensors. The application of 3D non-contact digitizers proposed in this paper allows for a quick and more complete monitoring of the health conditions of railways, also in combination with a proper procedure for automatic 3D inspection. The results of the experimental tests using 3D portable optical scanners on railways are compared with results obtained by a trolley instrumented with 2D contact sensors. The results demonstrate the effectiveness of the trolleys mounting 3D handheld optical digitizers with proper automated software inspection procedures.

## 1. Introduction

The inspection and monitoring of railway infrastructures are important issues for their sustainability and further maintenance. Currently, these diagnostic tasks are often repetitive, time consuming and, above all, they do not provide complete datasets to analyze the wear and damage progress over the rolling components, as most of them are based on human visual checks, the use of cut-outs and trolleys instrumented with 2D contact or optical measuring probes [1]. Moreover, the current inspection procedures on rolling components of railway lines imply large human involvement. For these reasons, the development of robotic or automated trolleys based on the use of 3D optical measuring instruments can be considered essential for diagnostics and the inspection of rail tracks, because they can provide data to get a complete scenario for the damage and they would allow the performance of inspection procedures with minimal human interventions, both during the measurement and data processing phases. In fact, 3D optical digitizers are becoming more and more useful to perform inspection and quality control in mechanics and biomechanics, thanks to their advanced performance due to technological innovations [2,3,4,5,6,7,8,9]. In [2], a cutting-edge procedure to assess wear, based on 3D optical digitizers, was presented and discussed. The procedure was further analyzed and applied in [3,4,6,7,8] to mechanical components of mixers, to knee prostheses and to cranial inserts, using new instruments and procedures properly developed [3,5,9]. A further application of 3D optical scanning devices in mobility issues is also related to the development of autonomous navigation systems, using obstacle and distance digitization as a guidance and applying different scanning principles, like laser or structured light triangulation [10,11].

3D optical scanners, differently from 2D and 3D contact measuring probes, allow the performance of inspections of components of every size and shape, without compromising their surface characteristics, which are often relevant during inspection procedures. Moreover, differently from 2D optical profilometers, 3D digitizers perform 3D complete and direct reconstructions of the samples under study, allowing inspection of every area of the surface and providing a wider overview and knowledge of the wheel–rail interaction (WRI). Furthermore, the use of 3D digitizers is one of the best methods to validate wear mathematical models developed to study the damage of rolling components [12,13,14,15,16,17,18,19,20,21,22]. 3D portable optical digitizers can create full digital models of components, avoiding dismounting them from the machine or from their exercise environment. Moreover, they can be mounted on robots or automated moving supports to perform 3D automatic inspection procedures. 3D automated and robotic inspection allows the performance of quality control on 100% of the samples in an exhaustive way. In fact, the complete digital model of the component under study can be superimposed with the 3D CAD (computer aided design) original project or with the scanned digital model of the undamaged or new component, and the inspection software gives the chance of detecting every single defect, unconformity or damage on the entire surface of the component [3,5,8,9,22,23,24,25].

A few studies have already demonstrated the efficiency and reliability of portable metrological 3D optical scanners for railways inspection. For example, in [1], the results of laboratory tests and on track tests were compared with results obtained with ultra-accurate contact probes and measuring trolleys already used for track control, showing the reliability and better efficiency of metrology optical 3D scanners and in particular portable metrology 3D laser scanners.

These instruments find a novel application on board of automated inspection trolleys for the study of WRI in combination with automatic software inspection procedures.

Some works in scientific literature report the use of 3D optical sensors or 3D imaging methods for railway and rolling component inspection [26]. In [27], a 3D scanner mounted on a robot was used for dimensional control of railway track slabs after production and before their delivery and installation.

In [28], a registration algorithm was proposed to process infrared images from an active thermography sensor for rail inspection. This system was able to detect subsurface defects by imaging methods and the aim of the algorithm was to overcome the issues due to the low contrast of infrared images taken on rolling components.

In [29], a 3D scanner, based on structured light technology, was proposed, and applied for inspection of the pantograph slide block.

The work described in [30] investigated the possibility of combining laser ultrasonic scanning detection and a hybrid intelligent method to classify artificial rolling contact fatigue defects in different depths.

In [31], the causes of image distortion captured by CCD cameras installed along the railway were analyzed.

In [32], a novel 3D laser profiling system (3D-LPS) was proposed, based on the integration of the laser profilometer Keyence LJ-V7000 with an odometer, an inertial measurement unit (IMU) and global position system (GPS), to capture the rail surface profile data. In this work, the system was mounted on an instrumented trolley for automatic defect detection of rails. When the carrier platform moved along the rail, it divided the measured object surface into many profiles. Thus, with this system, the 3D data of the rail surface were extracted from measurements of 2D profiles at every certain close distance. This means that the 3D surface was the result of a registration of different 2D data, processed with an adaptive iterative closest point (AICP) algorithm and approximating the surface data between two consecutive sections.

Differently from all the previous works, in this paper, 3D measurements are done by 3D laser sensors directly on tracks. 3D geometries are captured in real time without the need of reconstructing 3D surfaces by 2D profiles. In fact, the 3D laser scanner does not project just laser stripes but laser crosses, allowing the acquisition of 3D surfaces point by point. The demonstration of the feasibility of an automatic inspection trolley for railway lines and based on 3D portable handheld optical laser digitizers is the main objective of this paper, together with the experimental application of an automatic procedure for 3D inspection of tracks.

The novelties of the work presented in this paper are the application of 3D handheld optical metrology digitizers to a novel trolley, properly designed for single or twin control procedures of tracks and the development of a procedure for automatic 3D inspection, to be performed also during the 3D acquisition.

## 2. Current Instrumented Trolleys for Railways Inspection

Currently, ordinary diagnostics of rolling components is based on human visual inspections, to detect macroscopic damages. Measuring instruments are used periodically for specific diagnostics to assess the rail quality by checking a set of geometrical parameters on the rail tracks, such as track gauge, cant, crooked, 45° wear and vertical wear. The current measuring instruments used to check the damage of tracks due to the WRI are mounted on hand-pushed manual trolleys and are mainly based on the use of 2D contact or optical probes, able to acquire the rail profiles every certain distance travelled on the track and measuring rail corrugation and irregularities. These diagnostic trolleys are usually very slow and pushed by an operator, but there are others that are low-speed self-propelled. These methods allow the detection of a larger length of the line than manual surveys, but in any case, the length is limited by the system data acquisition speed, the capacity of the on-board capture system and the power system.

Based on the diagnostics’ result, corrective interventions can be planned, and useful-life can be forecast, leading also to obvious economic advantages. In fact, rail accurate measurement techniques are critical to guarantee transport security and reduce the maintenance cost.

**Figure 1:**
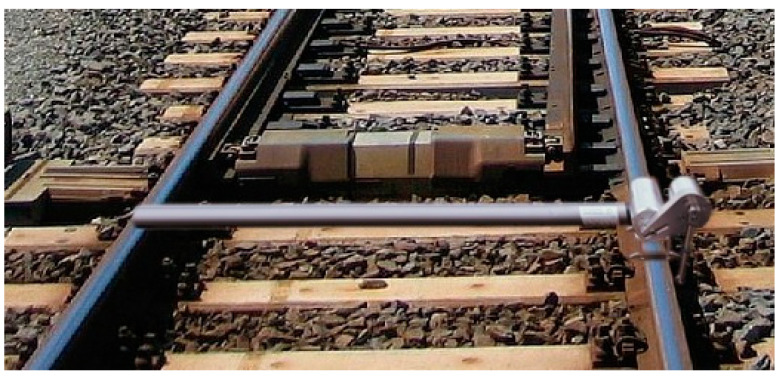
MiniProf Rail [35].

**Figure 2:**
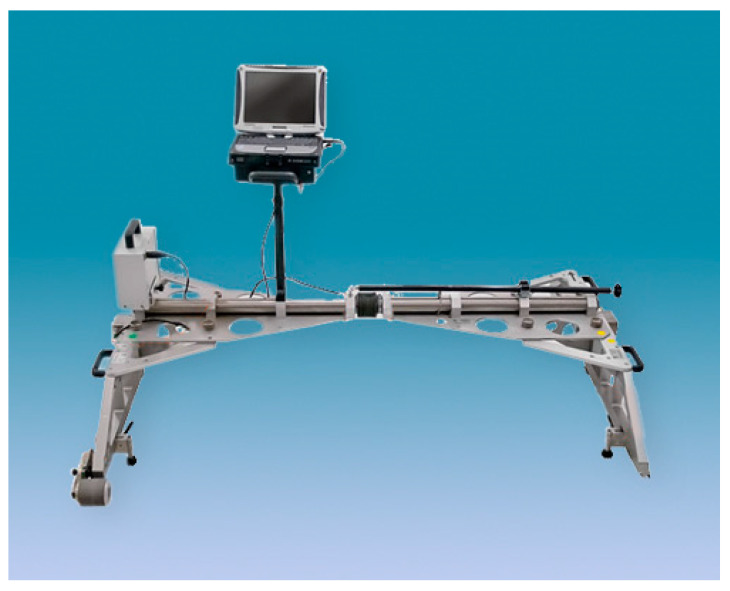
RMF-1100 [40].

**Figure 3:**
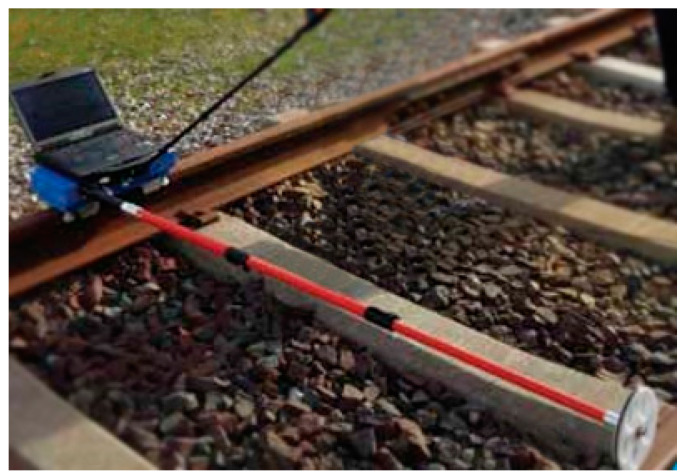
RailMeasurement’s CAT [41].

**Figure 4:**
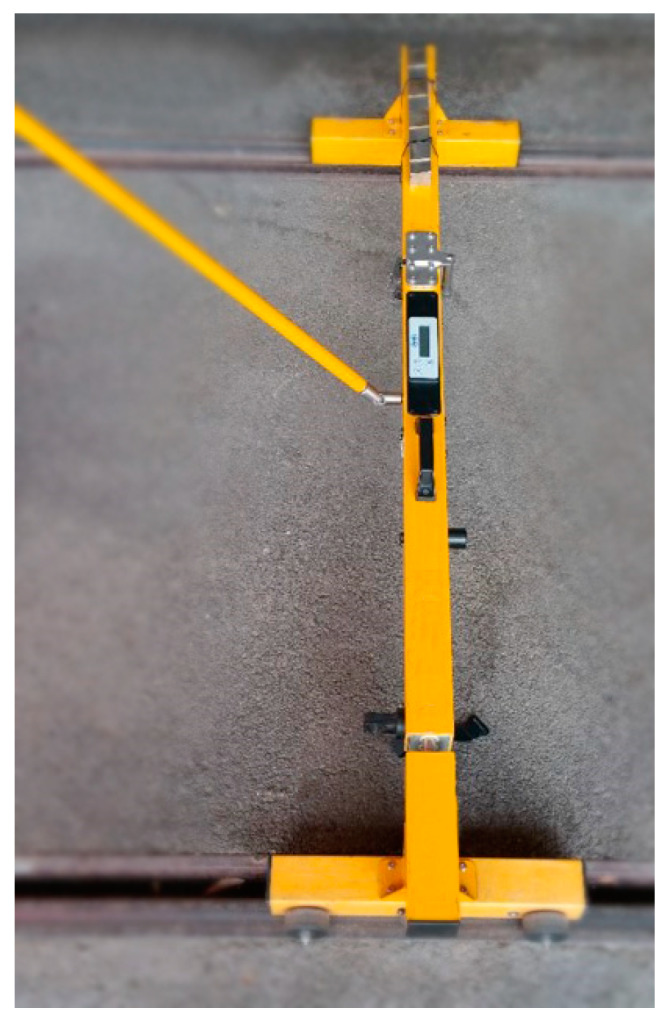
Amber [1,42].

**Figure 5:**
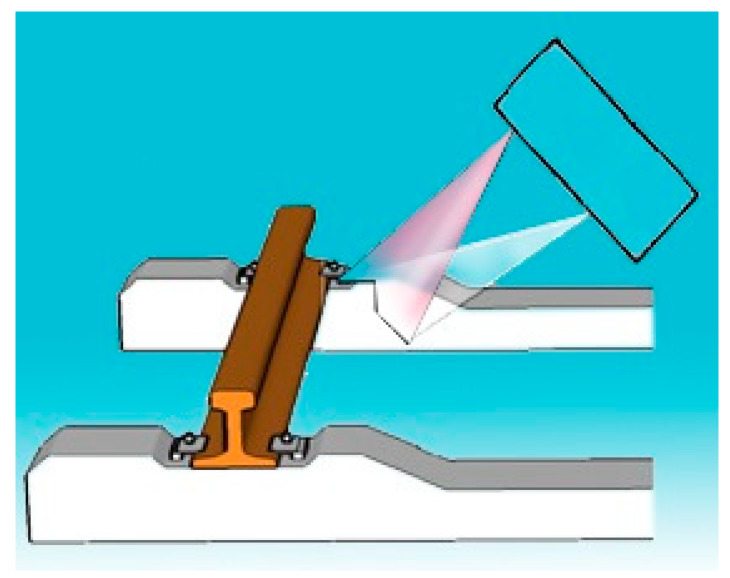
Structured light profilometer [43].

**Figure 6:**
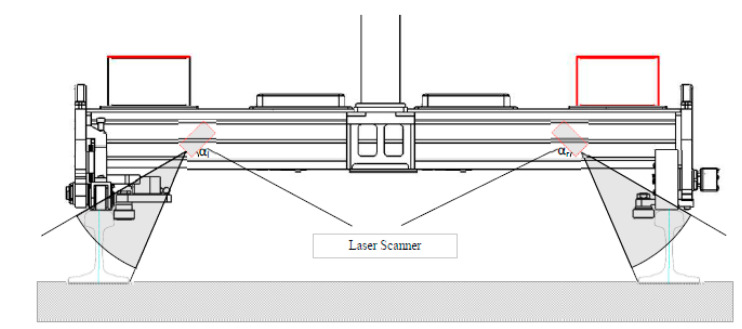
Inspection trolley for 3D reconstruction, based on laser profilometers [32].

At the state of the art, the main instruments used for evaluating the wear progress during the on-track WRI (Wheel–Rail Interaction) are:

- MiniProf Rail (Greenwood Engineering A/S, Brøndby, Denmark), (Figure 1). This is a tool to monitor the rail profiles, providing instant information on metal removal and grinding stone tilt. The instrument is a contact 2D profilometer, in which the sensing element is a magnetic wheel mounted on a shaft to be positioned in contact with the rail surface [14,15,16,19,20,33,34]. In the twin configuration, the opposite rail is considered as a reference by means of a telescopic rod [35].
- RMF-1100 (Vogel and Plötscher GmbH end Co. KG, Breisach, Germany), (Figure 2). The instrument is a continuous corrugation analysis trolley (CAT system) [17] which performs a real time continuous scanning of the rail longitudinal profile, and simultaneous measurements of the left and right rail. The sensing elements are measuring needles acting as a follower of a cam, where the cam is the longitudinal rail profile [36,37,38,39,40].

- CAT (RailMeasurement Ltd, Cambridge, UK), (Figure 3). RailMeasurement’s CAT is a hand-pushed trolley, instrumented for measuring rail corrugation and acoustic roughness on one rail, to show where rail reprofiling is needed. The trolley must go at a speed of 1 m/s [17,41]. It can be configured also as a bi-CAT system, so that a single operator can measure two rails simultaneously.
- AMBER (Geismar Ltd, Northampton, Northamptonshire, UK), (Figure 4). This is a rolling track gauge for recording the geometry of the track. It is hand-pushed, powered by a battery and designed for the continuous measurements of travelled distance, track gauge, cant and crooked. It is provided with an odometric wheel, which records the distance, and with side rollers for detecting the track gauge [1,42]. This device was used in this paper as a reference instrument to compare the results obtained from tests with the new proposed system. For this purpose, the performance parameters of AMBER are reported in Table 1.

- Optical 2D profilometer to measure the wear of the rails [43]. This is a fast and accurate structured light equipment for rail profile measurements to assess the wear progress over different rail sections. The inner rail profile is measured by a line structured light vision sensor (Figure 5). The sensor is not provided with a trolley.

- Optical 3D systems based on the use of laser profilometers. In this device, the 3D reconstruction is based on the acquisition of many 2D track’s profiles. In some cases, these systems are mounted on instrumented trolleys (Figure 6) as already seen in [32].

Many of the monitoring instruments previously presented, generally adopted for rail surveys, are not able to gather and provide a whole vision of the wear progress, being based on measurements of rail sections for every certain travelled distance. Furthermore, the optical ones, which are the most handling and effective, are not usually provided with a trolley and none of the present instruments allow for automated inspection procedures aided by specific software tools and avoiding large human interventions. Thus, the development of high accuracy, resolution and repeatability systems able to examine the condition of rails in their entirety to be used for automated inspection and monitoring, is a very important issue for maintenance tasks.

The development of such systems is proposed in the form of an industrial 3D portable optical scanner for metrology, to be flexible and installed on a trolley, which can be hand-pushed or motorized, introducing high efficiency, quality and repetitiveness in the inspection and monitoring of rails. The interoperability of these sensors and the possibility of automatizing the data processing and inspection throughout specific algorithms, may form a smart tool kit with powerful functions for inspection purposes. First, the experimental tests are reported to show the engineering feasibility of the system and interoperability of the trolley equipped with 3D handheld optical sensors that allow real time multiple acquisition and storage. The results of the experimental tests are compared with measurements performed with an AMBER trolley (Geismar Ltd, Northampton, Northamptonshire, UK) [1,42].

## 3. Design of New Trolleys for Railway Inspection Using 3D Portable Handheld Optical Sensors

In this work, two configurations of instrumented trolleys were studied to allow automatic 3D inspection of the railway tracks. Both the trolleys were hand-pushed and mounted 3D optical metrology sensors for capturing geometrical data of the tracks to be inspected. Furthermore, they were devised to accommodate a computer unit and a current generator for the power supply of all the technological components.

**Figure 7:**
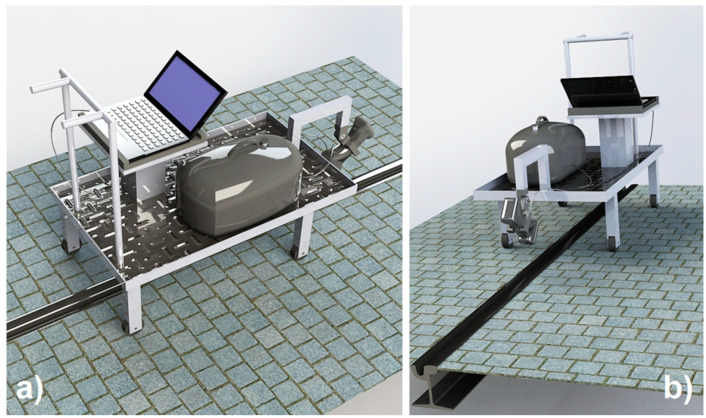
Instrumented trolley for single track automatic 3D inspection: (**a**) back-top view; (**b**) front-bottom view.

The first trolley was studied to inspect a single track by using a single 3D optical scanner (Figure 7).

**Figure 8:**
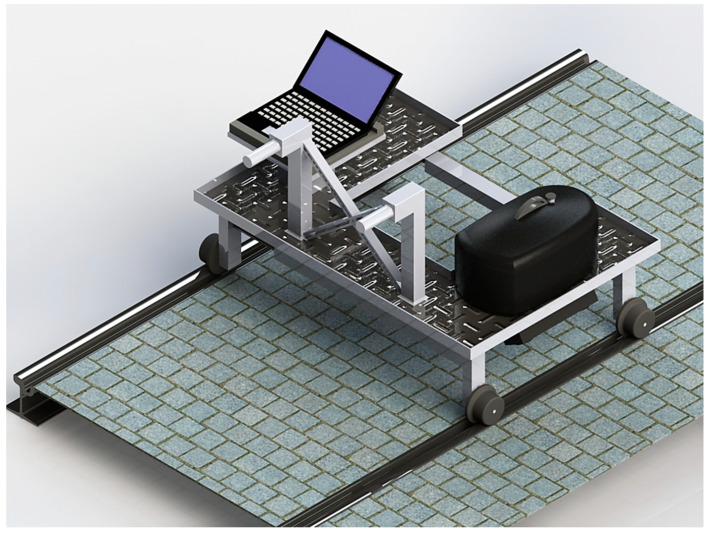
Twin instrumented trolley for complete 3D inspection of tracks.

**Figure 9:**
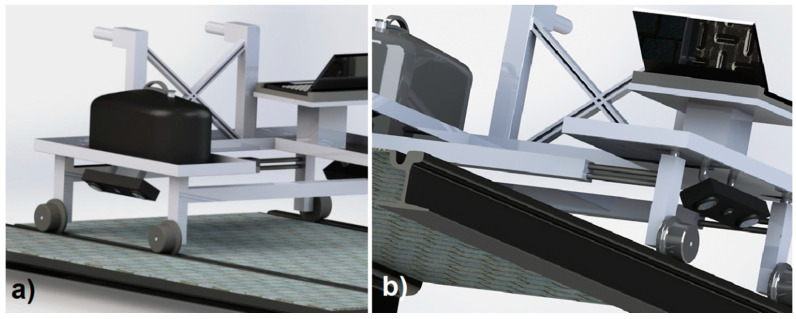
3D scanners on the trolley: (**a**) right side; (**b**) left side.

The second trolley was studied as a twin inspection trolley to allow detection of damages on both the tracks of a railway (Figure 8). For this reason, the 3D optical scanners mounted on this trolley were two, one for each side of the line (Figure 9).

The first solution resulted to be effective when the track and the ground had regular layout and for instance, it might be inappropriate for ballasts. On the contrary, the operation of the second studied trolley was devised to be free of issues related to ground irregularities. The twin trolley was conceived with adjustable distance of the wheels, in order to fit with different railway or tramway infrastructures.

The challenge of the development of both the trolleys was to allow 3D digital reconstructions of the tracks, avoiding the use of fixed references or absolute positioning and tracking devices. For this reason, both the trolleys were developed for mounting 3D portable optical digitizers. In fact, these instruments can acquire 3D geometrical data of objects without the need of fixed reference arms or tripods. They can be moved freely throughout the scanning volume and they reconstruct the 3D complete digital model of the objects by using different kinds of automatic alignments frame-by-frame.

On both the trolleys, the 3D scanning sensors were mounted on rotating or articulated arms. In the first trolley, the single 3D digitizer needed to be rotated around the pitch and roll axes and thus, it was mounted on an articulated two degrees of freedom (DOFs) arm. The twin trolley had one 3D sensor for each side of the track. In this case, the sensors just needed to roll.

**Figure 10:**
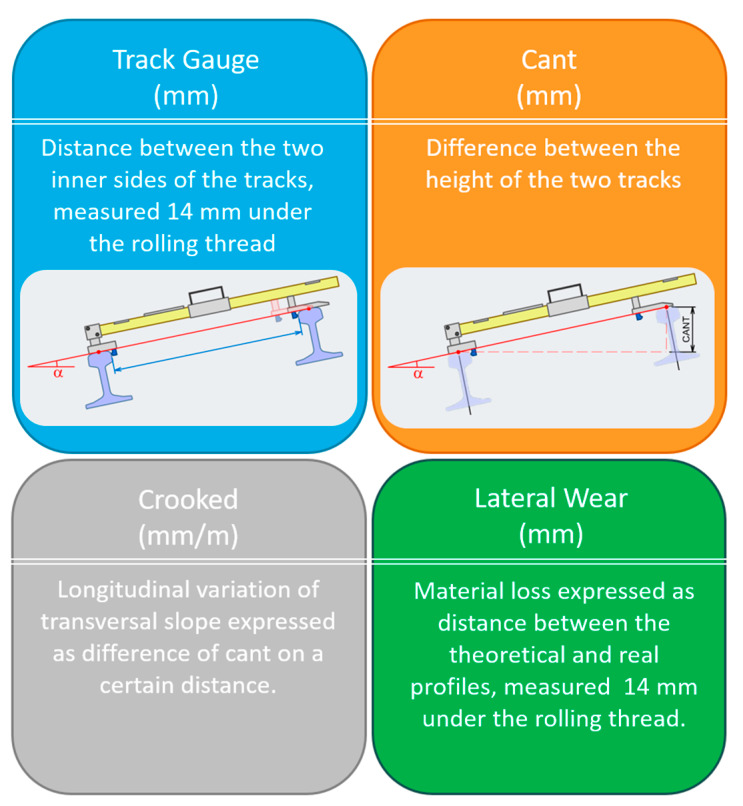
Geometric parameters to be measured by automated trolleys.

Both the trolleys were conceived to allow the measurement of a single track in all its parts, but the second configuration was also developed to allow the measurement of geometrical parameters also, like track gauge, cant, crooked and lateral wear (Figure 10), which need a twin configuration to be detected. A characteristic of the trolleys here described was also that they could mount different 3D portable handheld optical digitizers, even based on different scanning principles, like for example, structure light or laser triangulation.

**Figure 11:**
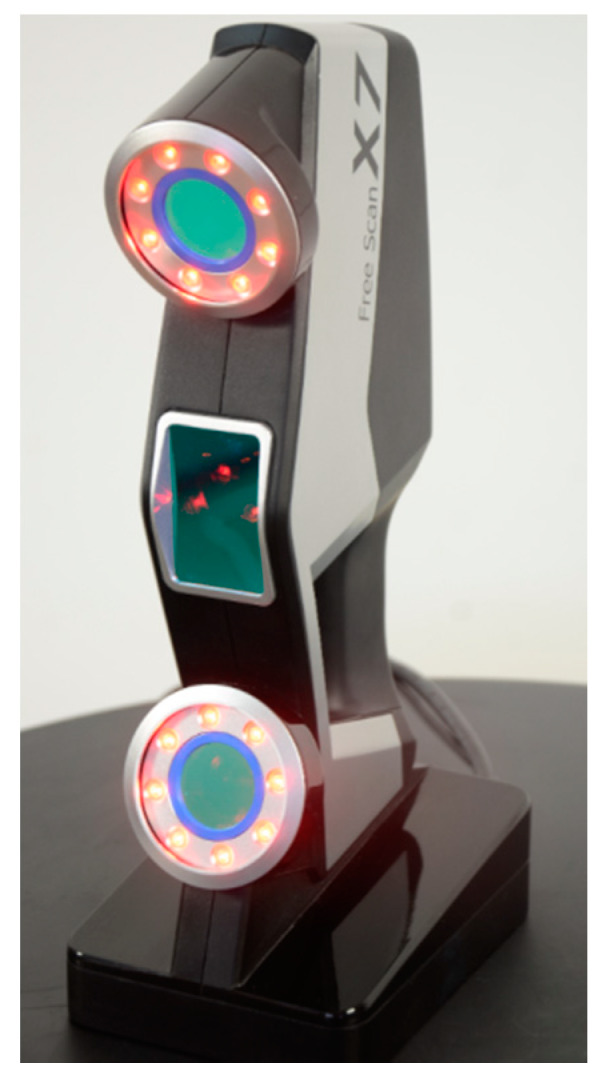
FreeScan X7 metrology 3D scanner.

The portable metrology 3D optical digitizer used for the feasibility study was FreeScan X7 (Shining 3D Tech. Co., Ltd., Hangzhou, China) (Figure 11). This 3D scanner uses 14 laser stripes and two cameras to digitize objects. Its performance parameters are: maximum resolution of 0.050 mm; single shot accuracy of 0.030 mm; volumetric accuracy of 0.020 + 0.060 mm/m; volumetric precision of 0.020 mm + 0.060 mm/m; scan speed of 480,000 points/s. The 3D digitizer is certified as a measuring instrument and provided with a metrology certificate, ensuring its performance. The device must be calibrated every year to guarantee its compliance with the declared parameters. The stand-off distance of the scanning device is 300 mm with a depth of field of 250 mm. Thus, the best distance between the 3D scanner and the track is 300 mm. This kind of instrument was chosen also because if there were distance differences due to track geometries, its depth of field would compensate the discrepancies.

Furthermore, this kind of scanner does not need any stabilization device or control to perform high quality 3D measurements in the presence of vibrations, as the stabilization of the results is achieved thanks to its internal reconstruction algorithm.

This digitizer allows real time 3D reconstructions and requires the use of physical targets, attached over the scene or over the object’s surface, to create a reference positioning model [2]. In the case of this work, the physical targets were attached on the ground around the tracks. The performance parameters of the chosen portable optical 3D digitizer were completely suitable for inspection of the rolling components.

When the 3D digitizer is mounted on the inspection trolley, results are not affected by speed in terms of accuracy, but they can be affected by speed in terms of density. A good trolley speed to allow a complete 3D scan of the whole geometries of the tracks is around 0.1 m/s. This is justified by the big and continuous amount of 3D data that the scanner can provide. The big amount of 3D data can also affect the speed of the subsequent inspection procedure, making it slow. To address this issue, in this work, two different solutions were proposed, one for the scanning procedure and one for the inspection phase. In fact, during the scanning session, the digitizing resolution was set to 2 mm, to make the 3D real time visualization more rapid and during the subsequent automated inspection procedure, the resulting mesh was further decimated, always preserving the shape and dimension of the track.

## 4. Automated 3D Inspection Procedure Development

The proposed automated digital 3D inspection is based on the use of real time scanning software such as FreeScan (Shining 3D Tech. Co., Ltd., Hangzhou, China), and a 3D inspection procedure to be implemented in inspection software like Geomagic Control X (3D Systems, Inc., Rock Hill, SC, USA). The phases of this technique are the following [2]:

1. Real time 3D scanning of the tracks.
2. Automatic 3D data optimization.
3. Real time inspection by comparison to track’s CAD models.

The results of prior tests performed in laboratory were used to develop each step of the track inspection procedure. In particular, a straight worn track portion was used as a specimen and it was detected by using the 3D optical laser scanner FreeScan X7 (Shining 3D Tech. Co., Ltd., Hangzhou, China). Different aligning and registration procedures were tested and compared using the mesh editing software Geomagic Wrap (3D Systems, Inc., Rock Hill, SC, USA) and the 3D inspection software Geomagic Control X (3D Systems, Inc., Rock Hill, SC, USA). The results were essential to set up the inspection procedure dedicated to railway infrastructures.

### 4.1. Track 3D Scanning

**Figure 12:**
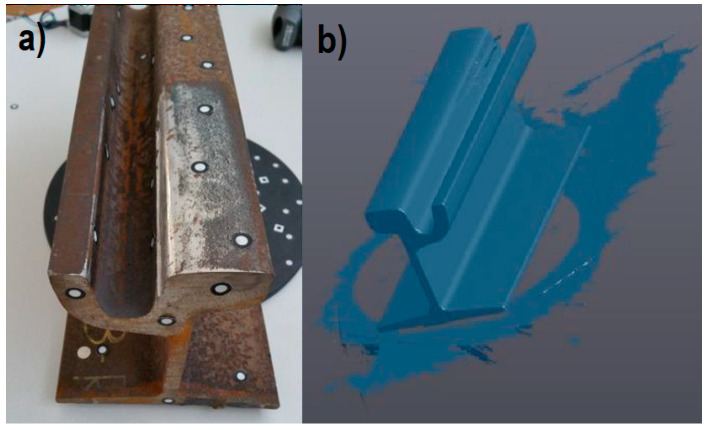
Sample worn track portion (**a**) and its 3D digital model (**b**).

The first phase of the proposed procedure is the 3D scanning session. This part of the procedure was validated by digitizing the sample worn track portion (Figure 12a) using the selected instrument and obtaining the 3D digital model shown in Figure 12b.

The complete sample model was generated in a 6 minute session, also considering the setup preparation and the application of the physical markers on the track’s surface.

The results were not affected by noise and the 3D model obtained by 3D scanning was compared section by section to three profiles obtained by applying the ultra-accurate 2D contact stylus Linear Height LH-600E/EG-Series 518 (Mitutoyo Corp., Kawasaki, Japan). The three sample sections were the initial, the final and the center section of the track portion. From this comparison, a perfect fitting between 3D and 2D results was highlighted and the 3D digitizing instrument was deemed suitable for the application. Thus, the 3D scanning phase was designed to be performed by moving the developed trolleys along the track to be inspected. The 3D real time metrology scanners mounted on the trolleys can capture the actual complete 3D digital model of the tracks by aligning all the scans using physical markers or geometrical features and ICP (iterative closest point) algorithms. The output model is a 3D point cloud or a triangular mesh.

### 4.2. 3D Data Optimization

The second phase of the inspection procedure is 3D data optimization. In this phase, it is crucial to preserve the size and shape of the model to ensure a reliable inspection result.For this reason, no standard mesh optimization options were used, but the mesh editing software was programmed to perform predetermined optimization functions to improve the quality of the 3D model without volumetric and shape alterations. In particular, detection and restoration of errors were performed by applying correction of non-manifold edges, self-intersections, highly creased edges, spikes, small holes and isolated patches. All the small holes were filled with the curvature criterion.

Thus, phase two can be performed by an automated inspection tool, properly programmed to be launched on the on-board computer, after the scanning session.

### 4.3. Track Inspection

The third phase is the proper inspection procedure, which is properly implemented into the commercial software to do automated operations and provide the inspection report.

#### 4.3.1. Alignment and Registration

The first operation of the automated inspection procedure is the alignment of the scanning model to the CAD original model of the track. This operation can be done by using different aligning algorithms.

**Figure 13:**
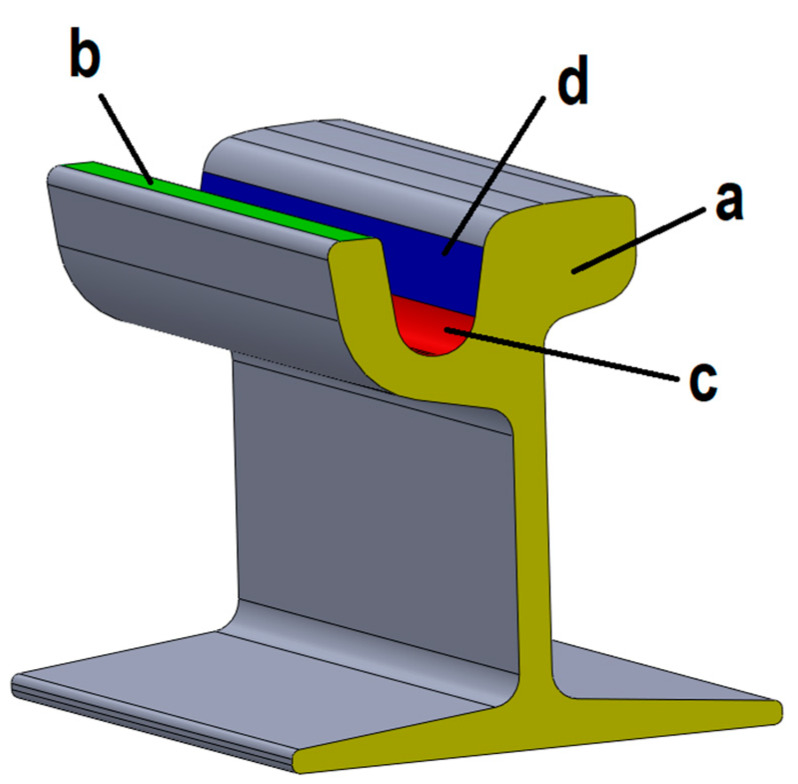
Automated inspection procedure: references for alignment. **a**: cross-section plane; **b**: counter rail; **c**: throat cylinder; **d**: flange.

Some experiments were done to choose the best registration technique to be applied for railway inspection, as in [1]. In particular, the 3D model obtained from the laboratory scanning session (Figure 12b) was aligned with the corresponding CAD model using three registration methods. With the first method (i), the mesh-to-CAD alignment was done, taking as a reference the cross-section plane (reference “a” in Figure 13), the counter rail plane (reference “b” in Figure 13) and the throat cylinder (reference “c” in Figure 13). With the second alignment (ii), the reference features were the counter rail plane, the cross-section plane and the flange (reference “d” in Figure 13). The third registration method (iii) was a best fit alignment.

Analyzing the results of the three aligning methods applied to the sample track portion, the first technique (i), based on the use of the cross-section plane, the counter rail plane and the throat cylinder as references resulted to be the most effective to detect the wear of rail infrastructures. In fact, these references represent the track’s areas which are not affected by wear or affected by wear to a lesser extent and for this reason, they are suitable to be used as reference unworn surfaces. In this way, the real wear phenomenon can be correctly detected onto the surfaces most affected by wear, like the rolling thread and the flange.

On the contrary, the method (ii) does not take into account the wear on the flange and can overestimate the wear on the rolling thread wear. The third technique (iii) is the worst aligning method to analyze wear of the tracks, because wear is not uniformly distributed over the entire surface of the tracks, and this generates misalignments.

Thus, the aligning method used in the proposed procedure is based on the use of the throat cylinder, the counter rail plane and the transversal plane as reference entities.

#### 4.3.2. Track’s Health Condition Inspection

The second operation of the automated inspection procedure is the comparison mesh to CAD, by using the cad model as a reference. Throughout this operation, the following data can be measured, calculated and displayed: a color map of deviations over the entire surface of the track, wear volume, wear rate, track gauge, cant, crooked and lateral wear for every certain travelled distance and their average values over an entire path. The procedure outputs also a set of cross-sections extracted from the color map for every specified certain distance. The value of the offset of every section from the start section can be set from the operator, as well as the values of the legend of the color map. The third operation of the inspection procedure is the automatic generation of the inspection report.

## 5. Experimental Tests, Results and Discussion

**Figure 14:**
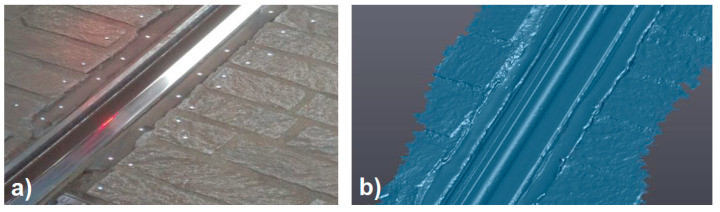
Experimental setup (**a**) and resulting 3D digital model (**b**).

Experimental tests were performed on a tramway track. The first step of the experiment was the scanning session of a part of the track with the selected metrology 3D scanner.A rectilinear track of 1.5 m length was digitized by using a positioning model made by physical markers applied on the ground around the track (Figure 14a).

**Figure 15:**
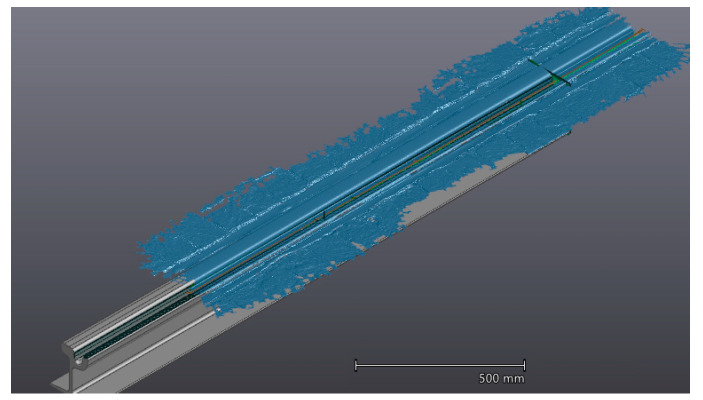
Automatic alignment.

**Figure 16:**
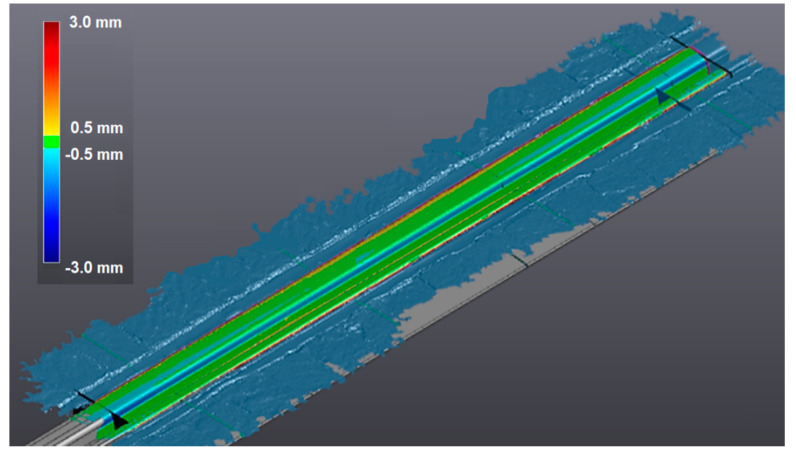
Color map of deviations.

The output digital 3D model of the track portion (Figure 14b) was used as input for the developed automatic inspection procedure. Figure 15 shows one step of the automatic alignment of the digital scanned model on top of the CAD model. Figure 16 shows one of the final results of the inspection: the color map of deviations. From this map, it was also possible to pick points manually to check punctual deviations.

**Figure 17:**
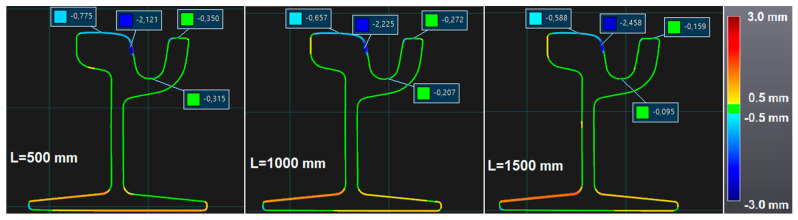
Inspected cross-sections.

The automated procedure gave also a set of cross-sections. Figure 17 shows three cross-sections automatically generated from the beginning section every 500 mm (L is the value of length). The labels in the picture were added manually to check some critical points.

All the measurements were performed by a single system, but the system was applied on both sides of the track and the average values of track gauge, cant and crooked, were calculated on 1.5 m length, analyzing the results of the inspection procedure. These results are listed in Table 2. Cant and track gauge could be measured by performing the inspection using 3D meshes of both sides of the tracks. Crooked could be measured performing the comparison between cants at different travelled distances.

All these results were compared with the results obtained by the AMBER trolley (Figure 4) [1,42], typically used for maintenance of the analyzed tracks.

The measures from the AMBER trolley are reported in the Table 3.

From the comparison of the results, one can observe that the average percentage difference in the measurement of the track gauge is 0.023% and there are no differences in the measurement of cant and crooked.

The benefit of applying this procedure is automatization in the software inspection process, the high quality and accuracy of results, and the possibility of collecting the complete 3D geometrical data for analysis of damages and defects of the rolling components, to gather a full view of the infrastructure health conditions.

In fact, results were compared on the basis of what the AMBER can check (cant, crooked and track gauge). Nevertheless, the system presented in this paper, resulting from the integration of a trolley, properly designed a 3D handheld metrology laser scanner and automated inspection procedures which can detect also other parameters, like wear rates, wear volumes, surface distribution of wear, local defects and damages due to wheel–rail contact and rolling contact fatigue like for instance squat and cracks, for providing a better solution with respect to current methods used on the studied tracks.

## 6. Conclusions

In this paper, a novel application of an automated procedure for 3D optical non-contact inspection of railway or tramway tracks was presented. Two different automated instrumented trolleys were studied, one for single track measurements and one for twin tracks inspection. Both trolleys were based on the application of portable optical metrology 3D scanners. A proper automatic 3D inspection procedure was studied and applied. Experimental tests were performed on a real tramway line with a portable handheld 3D laser scanner and with the studied automatic 3D inspection tool. 3D digital models of both sides of a track were acquired and processed by the inspection procedure. Track gauge, cant and crooked could be measured and the color maps of geometrical discrepancies, representing wear and surface damages, were displayed. As a further result, 2D profiles of the track could be studied by extracting them from the 3D digital model. A comparison with measurements performed by an AMBER trolley was performed. The results demonstrated the effectiveness of the trolleys mounting 3D handheld optical digitizers and highlighted the advantages of the automated measuring device and technique with respect to other devices in terms of accuracy of results, possibility of gathering a full view of the health conditions of the infrastructure and use of an automated inspection procedure. In fact, the presented inspection trolleys and technique give the opportunity of studying the rolling components in their entirety and not only in specific target points or surfaces, to enhance the knowledge of the railway health conditions and to plan more effective maintenance interventions. As a future work, the movement of the scanning arm can be developed, and the trolleys could be provided with electric drives to avoid the operator to push them. Further developments can be focused on new advancements of the proposed technique for rail track inspection, for instance, by providing scanning systems on board the vehicles, instead of trolleys. From this point of view, the involvement of absolute tracking devices would be preferable to avoid the use of physical markers.

## Figures and Tables

**Table 1:** AMBER measurement specifications.

Track Gauge Accuracy of the Measurement	From −20 mm to 50 mm ±1mm
Cant Accuracy of the measurement	±200 mm ±1mm from 0 to 200 mm
Crooked Accuracy of the measurement	±100 mm ±1.5 mm

**Table 2:** Geometrical parameters of the track resulting from the inspection procedure.

Parameter under Evaluation	L = 500 mm	L = 1000 mm	L = 1500 mm
Track gauge	1447.3 mm	1448.4 mm	1449.3 mm
Cant	1 mm	2 mm	1 mm
Crooked	0.1 %		

**Table 3:** Geometrical parameters of the track resulting from the AMBER’s measures.

Parameter under Evaluation	L = 500 mm	L = 1000 mm	L = 1500 mm
Track gauge	1447 mm	1448 mm	1449 mm
Cant	1 mm	2 mm	1 mm
Crooked	0.1 %		
